# Extraction Optimization of Astragaloside IV by Response Surface Methodology and Evaluation of Its Stability during Sterilization and Storage

**DOI:** 10.3390/molecules26082400

**Published:** 2021-04-20

**Authors:** Lin Xu, Kongjiong Wei, Jiaolong Jiang, Lianfu Zhang

**Affiliations:** 1 School of Food Science and Technology, Jiangnan University, Wuxi 214122, China; linxu7813@gmail.com; 2 Gansu Longcuitang Nutrition Food Corp., Ltd., Lanzhou 730046, China; lct_kongjiong@163.com (K.W.); lct_jiaolong@163.com (J.J.)

**Keywords:** astragaloside IV, response surface methodology, stability, heat sterilization, storage

## Abstract

Radix Astragali is referred to as a variety of food-medicine herb, and it is commonly applied as Traditional Chinese Medicine (TCM). However, it is extremely difficult to extract its bio-active compounds (astragaloside IV) and apply it in food processing efficiently, which restricts its practical applications. In this study, the conditions required for the extraction of astragaloside IV were optimized by following the response surface methodology. More specifically, ammonia with a concentration of 24% was used as an extracting solvent, the solid–liquid ratio was 1:10 (w:v); the Radix Astragali was soaked at 25 °C for 120 min in advance and then stirred at 25 °C for 52 min (150 rpm) to extract astragaloside IV. This method promoted the transformation of other astragalosides into astragaloside IV and replaced the traditional approach for extraction, the solvent reflux extraction method. The yield of astragaloside IV reached the range of 2.621 ± 0.019 mg/g. In addition, the stability of astragaloside IV was evaluated by detecting its retention rate during sterilization and 60-day storage. As suggested by the results, the astragaloside IV in acidic, low-acidic, and neutral solutions was maintained above 90% after sterilization (95 °C and 60 min) but below 60% in an alkaline solution. High temperature and short-term sterilization approach is more appropriate for astragaloside IV in an alkaline solution. It was also found out that the astragaloside IV obtained using our method was maintained over 90% when stored at room temperature (25 °C), and there was no significant difference observed to low temperature (4 °C) in solutions regardless of acidity.

## 1. Introduction

**Figure 1:**
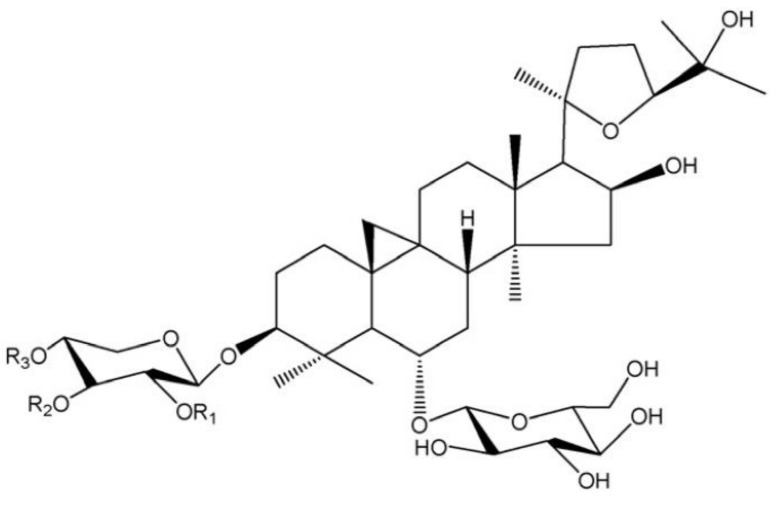
The chemical structures of astragaloside I, II, and IV [14]. Astragaloside I: R1 = R2 = Ac, R3 = H; astragaloside II: R1 = Ac, R2 = R3 = H; astragaloside IV: R1 = R2 = R3 = H.

Radix Astragali (Huangqi), a widely-known traditional food–medicine herb [1], has been approved to be applied as food ingredients by the National Health Commission of China and the European Food Safety Authority (EFSA) Panel on Nutrition [2]. However, the most common application of Radix Astragali is water-soaking [3,4,5], without considering efficiency and the stability of the main bioactive compound [6], astragaloside IV. Astragaloside IV is considered the herb’s major bioactive component [7] for its pharmacological and pharmacokinetic properties, such as its anti-inflammatory effect [8], anticancer activity [9], and neuroprotective activity [10]. At present, astragaloside IV is recognized by the Chinese Pharmacopoeia (2015 version) as a qualitative control standard. However, astragaloside IV is disadvantaged by a poor solubility (both in water and organic solvents) and low content, which makes the extraction of it far from efficient [11]. Additionally, it is possible for other astragalus saponins in Radix Astragali, especially astragaloside I and astragaloside II [12], to be transformed into astragaloside IV in certain conditions. The chemical structures of astragaloside I, II, and IV were shown in Figure 1. The addition of lye into the solvent can help hydrolyze the acetyl base to fall off on the xylan terminal chain and convert other astragalus saponins into astragaloside IV easily [13], thus improving the yield of astragaloside IV.

There are various influencing factors in the transformation of other astragalus saponins into astragaloside IV, e.g., the type and concentration of the alkaline solution, the solid–liquid ratio, the soaking time, the extraction time, and the extraction temperature. On the one hand, too much alkaline and a too-high extraction temperature can cause the saponin structure to collapse and reduce the yield. Mei Xiaodan et al. [15] explored the yield of astragaloside IV extracted for 30 min using a 2.5–25% ammonia and 1% sodium hydroxide solution, but this failed to achieve any further optimization. The problem lies in how to remove the alkaline in a non-toxic way. In some other studies [12,15], a macroporous resin elution was used to remove alkaline after extraction. However, this method consumed too much energy and was toxic due to the risk of particle residue. Thus, this method is unsuited to food processing. Therefore, it is essential to identify suitable alkaline species and determine favorable extraction conditions.

In addition, the instability of the astragaloside IV extract, especially the alkaline extract, is an urgent problem to be solved. Firstly, astragaloside IV is a natural extract, and the stability of natural extracts is a matter of great concern. Because the instability of natural extracts can make products unstable and cause curative effects, it is difficult to provide stable products for consumers. The content of astragaloside is easily affected by temperature and acidity, but the effect is not clear. Secondly, as a new resource food raw material, there is little research on the stability of Radix Astragali or astragaloside IV in food processing. Zheng Na et al. [16] researched the retention rate of astragaloside I, II, and IV standard in pH 2.0 and 6.8 solutions within 80 h, but these were far from application scenarios. In order to simulate the acidity of the digestive system, Feng Xiaoquan et al. [17] conducted an investigation into the content of the astragaloside IV in a 0.1 mol·L^−1^ HCl solution and 8% ammonia, which led to the finding that the content of astragaloside IV is affected by acidity. Additionally, these limited studies were based on the astragaloside standard rather than the extract system. Thirdly, exploring the stability of an extract obtained using this method can provide guidance on the subsequent processing of Radix Astragali to some extent, e.g., the improvement of sterilization or storage conditions. According to the level of acidity, food is categorized into acidic, low-acidic, neutral, and alkaline foods, with different types of food requiring different sterilization conditions [18]. It is thus necessary to investigate the change in the retention rate of astragaloside IV after lye extraction under different conditions of sterilization processing and storage, which will contribute not only to ensuring high quality and safe products for consumers but also to widening the scope of applications for Radix Astragali. In this paper, the relationship between the thermal sterilization stability and acidity of astragaloside IV was investigated, and suggestions were put forward to ensure the stability of astragaloside IV in processing. Moreover, there is still no research on the retention rate of astragaloside IV extracted by lye after sterilization during storage.

Different from other studies [12,15], this study features the introduction of the pretreatment process before the extraction of astragaloside IV, which shortens the extraction time, reduces energy consumption, and improves extraction efficiency. Additionally, an ammonia solution is taken as the solvent, because it is easy to remove by vacuum evaporation and the need to use macroporous resin elution methods (which carries the risk of macroporous resin residue and is prohibited from food processing) is avoided. The response surface method is frequently used to analyze multiple variables responding to one or more responses [19,20]. In this study, the extraction conditions were optimized for the astragaloside IV by following the response surface methodology. At the same time, an exploration was conducted into the stability of astragaloside IV during the common sterilization processes in acidic, low-acidic, neutral, and alkaline conditions, as well as in 60-day storage. In addition to the yield of astragaloside IV, astragaloside I and astragaloside II were also detected by ultra-performance liquid chromatography–tandem mass spectrometry (UPLC–MS/MS) [21] throughout the process (extraction, sterilization, and storage) in order to establish whether the transformation of astragaloside IV would be reversed.

## 2. Materials and Methods

### 2.1. Materials

Radix Astragali was collected from Longxi, Gansu Province in China. After being cleaned and dried in a heating-air drying oven at about 40 °C until constant weight, the raw materials were smashed and sieved through a 6-mesh sieve. The powder was sealed in a PE bag and then kept at −20 °C for later use. Astragaloside I, astragaloside II, and astragaloside IV standard compounds were purchased from Shanghai yuan ye Bio-Technology Co., Ltd. (Shanghai, China) with a purity of >98%. Methanol (HPLC grade), acetonitrile (HPLC grade), methanoic acid (HPLC grade), ammonium hydroxide, and ethanol were sourced from Sinopharm Chemical Reagent Co., Ltd. (Shanghai, China). Ultrapure water was used for the preparation of all solutions.

### 2.2. Quantitation of Astragaloside I, Astragaloside II, and Astragaloside IV

The quantitation of astragaloside was performed using the method proposed by Qi L.W. et al. [14] with some modifications. UPLC–MS/MS quantitated all astragalosides with a UPLC–TQD system in the + ESI mode. The purified sample (1 μL) was injected into an ACQUITY C18 (1.7 μm, 2.1 × 100 mm) column with a gradient elution comprised of eluents A (100% acetonitrile) and B (0.1%, *v/v* formic acid in water) at 0.3 mL/min. The column temperature was maintained at 45 °C, and the mobile phase elution procedure was as follows: 0–1 min, 20% A; 1–10 min, 20–100% A; 10–11 min, 100% A; 11–12 min, 100–20% A; and 12–14 min, 20% A. The MS/MS analysis was conducted at 700–900 *m*/*z*, with N2 (>99.99%) as the drying gas and the flow set to 500 L/h. The source block temperature and desolvation temperature were 100 and 400 °C, respectively. The capillary voltage was set to 3.0 kV, and the detector voltage was set to 1800 V. Mass Lynx software was applied for data and graph analysis. The quantitation of astragalosides was performed using a calibration curve of the corresponding purified astragalosides.

### 2.3. Quantitative Validation Calibration Curves

#### 2.3.1. Calibration Curves, Limits of Detection (LOD) and Quantification (LOQ)

In order to plot the calibration curves, methanol stock solutions containing astragaloside I, II, and IV were prepared and diluted to appropriate ranges of concentration. The quantitation of astragaloside I, astragaloside II, and astragaloside was identified following Section 2.2., while the linear regression equations of astragaloside I, II, and IV were calculated with the mass concentration of astragaloside I, II, and IV as the horizontal coordinate and the peak area as the vertical coordinate. Under the present chromatographic conditions, the LOD and LOQ were determined at the signal-to-noise ratios (S/Ns) of 3 and 10, respectively.

#### 2.3.2. Precision and Accuracy

With 4 g of sample accurately weighed, the sample solution was prepared following Section 2.4., while the retention time and peak area of each substance were determined 6 consecutive times according to Section 2.2. within the space of 1 d. The intra-day precision was calculated using relative standard deviation (RSD%). Similarly, the same process was repeated 6 times per day for 3 consecutive days in order to calculate the inter-day precision by using RSD%. Additionally, 1 g of the samples with a known content of astragaloside I, II, and IV was accurately weighed, and this was repeated 6 times. With astragaloside I, II, and IV at concentrations of 26.67, 13.33, 8.33 μg/mL, respectively, 1 mL of a standard solution was added. The sample solution was prepared according to Section 2.4., the content of each substance was determined according to Section 2.2., and the recoveries were calculated (%).

### 2.4. Single-Factor Experiments of the Extraction Process

The control variable method was adopted to investigate the potential impact of ammonia concentration, solid–liquid ratio, soaking time, extraction time, extraction temperature, and extraction time on the yield of astragaloside IV, with the separation of astragaloside I, II, and IV performed in accordance with the work of Shaw et al. PLoS one, Published: 29 August 2012, https://doi.org/10.1371/journal.pone.0043848 [22]. To be specific, about 3 g of astragalus powder were accurately weighed, while a certain volume of an ammonia solution of a certain concentration was added and soaked at 25 °C for some time. Additionally, an oscillating extractor was used at the given temperature at 180 rpm for some time. Then, the solution was concentrated by vacuum-rotary evaporation at 45 °C. The residue was dissolved by 10 mL of water and extracted by 40 mL of n-butanol saturated with water. After extraction was performed four times, the extraction of n-butanol saturated with water was combined. The solution was concentrated to 10 mL by vacuum-rotary evaporation at 45 °C and filtered using a filter (0.22 lm; Whatman, Maidstone, UK), suitable for UPLC–TQD analysis. The astragaloside IV was determined, and the yield was calculated using Equation (1), with the analysis of parallel measurements done three times:

The astragaloside IV yield = Weight of astragaloside IV (mg)/Weight of Radix Astragali (g)(1)

The specific extraction conditions were set as follows:

#### 2.4.1. Ammonia Concentration 

The ammonia concentration was set to 0%, 10%, 20%, 30%, and 40%. The solid–liquid ratio, soaking time, extraction time, extraction temperature, and number of times of extraction were set to 1:10, 30 min, 30 min, 20 °C, and 1, respectively.

#### 2.4.2. Solid–Liquid Ratio 

The solid–liquid ratio was set to 1:3, 1:10, 1:20, 1:30, and 1:40. The ammonia concentration, soaking time, extraction time, extraction temperature, and number of times of extraction were set to 20%, 30 min, 30 min, 20 °C, and 1, respectively.

#### 2.4.3. Soaking Time

The soaking time was set to 0, 60, 120, 180, and 240 min. The ammonia concentration, solid–liquid ratio, extraction time, extraction temperature, and number of times of extraction were set to 20%, 1:10, 30 min, 20 °C, and 1, respectively.

#### 2.4.4. Extraction Time 

The extraction time was set to 0, 60, 120, 180, and 240 min. The ammonia concentration, solid–liquid ratio, soaking time, extraction temperature, and number of times of extraction were set to 20%, 1:10, 60 min, 20 °C, and 1, respectively.

#### 2.4.5. Extraction Temperature

The extraction temperature was set to 0, 20, 40, 60, and 80 °C. The ammonia concentration, solid–liquid ratio, soaking time, extraction time, and number of times of extraction were set to 20%, 1:10, 60 min, 30 min, and 1, respectively.

#### 2.4.6. Extraction Times

After the first-time extraction, the solid was recycled and extracted a second time and a third time. The ammonia concentration, solid–liquid ratio, soaking time, extraction time, and extraction temperature were set to 20%, 1:10, 60 min, 30 min, and 20 °C, respectively.

### 2.5. Response Surface Method to Optimize Extraction Conditions

From the single-factor experiments conducted on the extraction process, it was known that the extraction temperature caused a reduction to the yield of astragaloside. The number of times of extraction times made no significant difference to the yield of astragaloside. Therefore, ammonia concentration (A), solid–liquid ratio (B), soaking time (C), and extraction time (D) were selected as the factors to investigate, while the yield of astragaloside IV was treated as the investigative indicator. Additionally, the Box–Behnken approach was used to design a four-factor and three-level response surface experiment. Table 1 shows the factors and levels. The solution was concentrated to 10 mL by means of vacuum-rotary evaporation and filtered using a filter (0.22 lm; Whatman, Maidstone, UK) for subsequent UPLC–TQD analysis. The optimization conditions were predicted using a second-order polynomial equation.

### 2.6. The Retention Rate of the Astragaloside IV Under Different Sterilization Conditions

According to the conditions required for response surface optimization, the extract of astragaloside IV was obtained at a 24.15% ammonia concentration, a solid–liquid ratio of 1:10, a soaking time of 120 min, and an extraction time of 52 min. The pH value of the solution at 3, 5, 7, and 9 was adjusted to simulate acidic, low-acidic, neutral, and alkaline food environments, respectively. The solution was simulated at a given sterilization temperature and time. The treated solution was treated using the methods in Section 2.2 so as to detect the changes of astragaloside I, astragaloside II, and astragaloside IV in the solution. The specific sterilization conditions are shown as follows: heating at 85 °C for 30, 40, 50, and 60 min; heating at 90 °C for 30, 40, 50, and 60 min; heating at 95 °C for 30, 40, 50, and 60 min; heating at 85 °C for 30, 40, 50, and 60 min; heating at 100 °C for 15 and 30 min; heating at 105 °C for 15 min; and heating at 120 °C for 10 min. The astragaloside IV content was determined by UPLC-TQD, the retention rate was calculated using Equation (2), and parallel measurements were analyzed three times:

The retention rate of astragaloside IV (%) = Weight of astragaloside IV after sterilization (mg)/Weight of Astragaloside IV before sterilization (mg) × 100%(2)

### 2.7. The Retention Rate of Astragaloside IV in Different pH Value Solutions Stored at 4 or 25 °C

After sterilization, solutions with different pH values were vacuum-sealed in aluminum foil packaging bags and then stored at 4 and 25 °C for 60 days, which is common during the transportation and storage of food and beverages [23]. During the period, the content of astragaloside IV was detected to calculate the retention rate through regular inspection. Moreover, the contents of astragaloside I and astragaloside II were detected to determine whether the transformation of astragaloside IV was reversed.

### 2.8. Statistical Analysis

A response surface data analysis was conducted using the Design Expert. V8.0.6 (Minneapolis, MI, USA) software, and other statistical data analyses were carried out using GraphPad Prism 8.0.2 (GraphPad Software Inc., San Diego, CA, USA). In addition, a *t*-test was performed to make unpaired observations, based on which the statistical evaluation of differences was conducted, with a *p*-value less than 0.05 treated as statistically significant. All tests were carried out through three parallel operations, with the results expressed as mean ± SD.

## 3. Results and Discussion

### 3.1. Identification of Astragaloside I, Astragaloside II, and Astragaloside IV by UPLC–MS/MS

**Figure 2:**
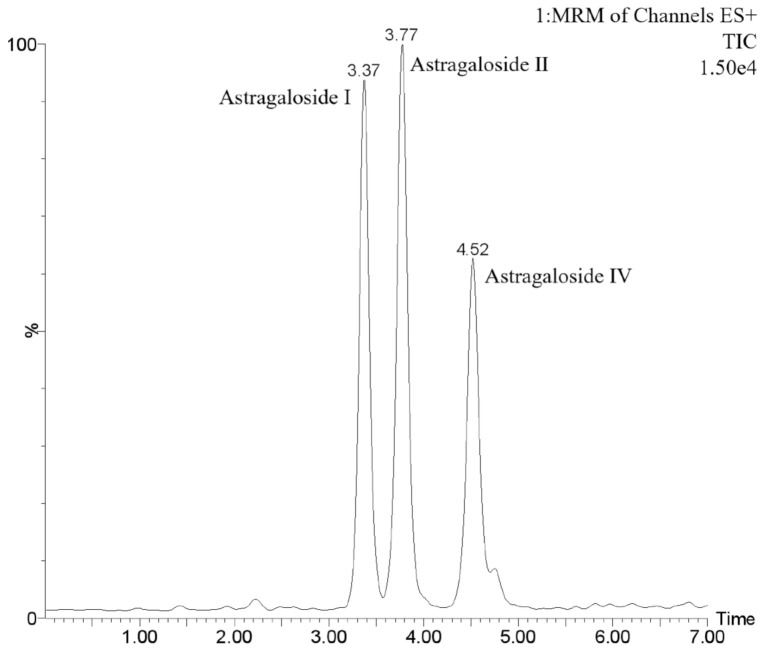
The UPLC–TQD chromatogram of astragaloside I, astragaloside II, and astragaloside IV.

Figure 2 shows the UPLC–MS/MS chromatogram of astragaloside I, astragaloside II, and astragaloside IV. Table 2 lists the MRM parameters of astragaloside I, astragaloside II, and astragaloside IV.

#### Quantitative Validation

As shown in Table 3, the linearity of analytical response was acceptable with the correlation coefficients higher than 0.99, thus providing a dynamic range of about two orders of magnitude. The LODs of astragaloside I, astragaloside II, and astragaloside IV fell within the range of 0.002–0.006 μg, the LOQs varied from 0.009 to 0.024 μg, the intra-day RSDs ranged between 1.4% and 3.7%, the inter-day RSDs varied between 1.1% and 1.8%, and the recoveries exceeded 94%. Suitable for the detection of astragaloside I, astragaloside II, and astragaloside IV, this method is effective in shortening the detection time and improving efficiency.

### 3.2. Single-Factor Experiments

#### 3.2.1. Ammonia Concentration

**Figure 3:**
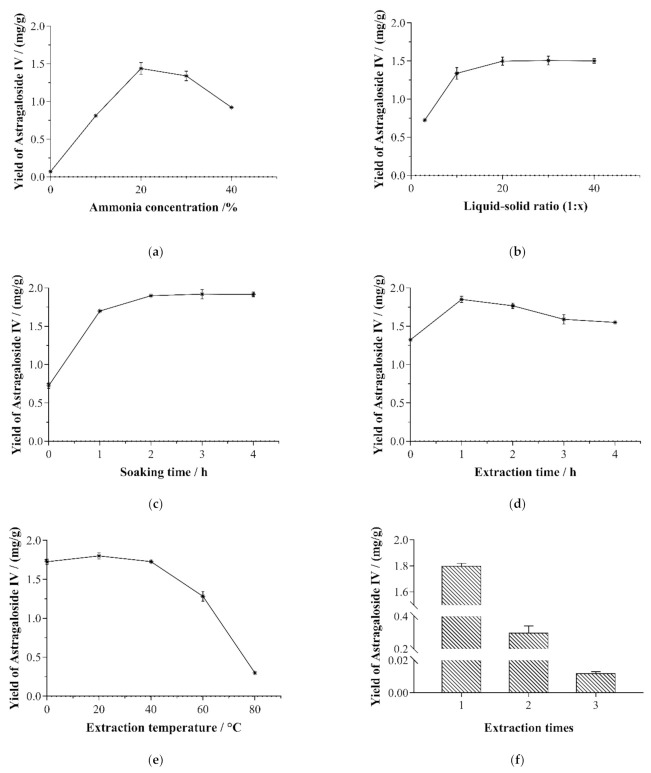
The effects of ammonia concentration (**a**), solid–liquid ratio (**b**), soaking time (**c**), extraction time (**d**), extraction temperature (**e**), and extraction time (**f**) on the yield of astragaloside IV in single-factor experiments of the extraction process.

Ammonia concentration is one of the most critical influencing factors in the content and yield of saponins. As shown in Figure 3a, the yield of astragaloside IV first increased and then decreased when the ammonia concentration varied between 0% and 40%. In comparison, higher and lower ammonia concentrations caused reductions to the content and yield of saponins. That is to say, an insufficient alkali content will lead to an incomplete conversion between astragalus saponins, but an excessive amount of ammonia is suitable to hydrolyze astragalus saponins [15]. According to the results, the ammonia concentration was set to the range of 20–30% for extracting the astragalus saponins.

#### 3.2.2. Solid–Liquid Ratio

Increasing the liquid-to-solid ratio is an effective solution to improving the content and yield of astragaloside IV by adjusting the concentration gradient in and out of cells in the solution. In this study, the water-to-solid ratios of 3, 10, 20, 30, and 40 were investigated. As shown in Figure 3b, as the water-to-solid ratio rose sharply from 3 to 20, and the content and yield of astragaloside IV increased accordingly, which was possibly because the rising water-to-solid ratio increased the diffusion of solvent in cells and enhanced the desorption of astragaloside IV from cells [24]. When the water-to-solid ratios were set at 30 and 40, however, the components were completely dissolved into the extract. As a result, the variation in the yield of astragaloside IV was found to be insignificant (*p* < 0.05). Thus, the water-to-solid ratio was set to 10–20% for the extraction of astragalus saponins.

#### 3.2.3. Soaking Time

Then, an investigation was conducted into the impact of soaking time on the yield of astragaloside IV, with the extraction time sets to 0, 1, 2, 3, and 4 h. According to Figure 3c, as the soaking time was extended, the yield of astragalus saponins first increased rapidly and then slowed down. This was because a longer soaking time was conducive to the transformation of astragalus saponins at the initial stage of soaking [15]. Other astragalus saponins could be transformed into astragaloside IV immediately. With the further extension to the soaking time, the transformation process got close to completion, and the effect of increasing the yield of astragaloside IV was made less significant than before. In order to reduce cost, the soaking time was determined as 60–120 min in the following response surface methodology experiments. 

#### 3.2.4. Extraction Time

The effects of the extraction time on the yield of astragalus saponins were then investigated with the extraction times set to 0, 1, 2, 3, and 4 h. As shown in Figure 3d, the yield of astragalus saponins first increased and then declined as the extraction process was prolonged. The reason behind it was that, at the initial stage of extraction, a longer extraction time was favorable to dissolving astragaloside IV and other polar compounds in the solvent. However, with an extension to this period, it was easy for astragaloside IV, as a kind of triterpenoid saponins, to be decomposed [25]. The concentration of other ingredients in the solvent reached the equilibrium, so the change of dissolution was limited. Additionally, there might have been some adverse reactions taking place that reduced the yield. Additionally, over the course of extraction, the increase of suspension viscosity was unfavorable to improving the extraction efficiency, and astragaloside IV could hardly be extracted from cell debris [26]. Based on the results, the extraction lasting 0–1 h was judged as effective in achieving a higher yield of saponins. Therefore, 0, 0.5, and 2 h were treated as the optimal conditions in the following response surface methodology experiments.

#### 3.2.5. Extraction Temperature

On the one hand, increasing the extraction temperature can improve the efficiency of transformation for astragalosides. On the other hand, a high temperature could accelerate the collapse of astragaloside IV [15,25]. As shown in Figure 3e, the yield of astragaloside IV slowly increased with the rise of extraction temperature up until 40 °C and then decreased. In order to reduce costs, the extraction temperature was determined as 20 °C.

#### 3.2.6. Extraction Times

Figure 3f shows the impact made by the number of times of extraction on the yield of astragaloside IV. It could be seen from the figure that the second-time extraction led to as little as 0.326 ± 0.017 mg being extracted. This was due to the presence of some astragaloside residues in the gap of astragalus powder during the extraction process. Additionally, production costs were continuously on the rise. Therefore, the number of times of extraction was set to two as the optimal condition in the following experiments. Moreover, for the second-time extraction, the liquid-to-solid ratio was set to 1:1 for cost reduction.

### 3.3. Response Surface Methodology

#### 3.3.1. Response Surface Experimental Design and Response

As shown in Table 1 and Table A1, there were twenty-nine tests conducted under different conditions, as designed using the response surface method. Moreover, Table A1 lists the experimental results corresponding to each test combination. The yields of astragaloside I and astragaloside II were also detected at the same time as astragaloside IV, as shown in Appendix A,Table A1. Moreover, Design-Expert, version 8.6 (Stat-Ease Inc., Minneapolis, MN, USA) was applied to calculate the multiple linear regression equation according to the response value and the experimental conditions. The software was also used to calculate the regression coefficients. The fitted equations to predict the yield of astragaloside IV from the Radix astragali are given as follows:

The yield of astragaloside IV = 2.19 − 0.36 × A + 8.167 × 10^−3^ × B + 0.26 × C + 0.091 × D−0.013 × A × B + 0.085 × A × C − 0.040 × A × D − 0.12 × B × C + 0.038 × B × D + 0.045 × C × D − 0.86 × A^2^ − 0.012 × B^2^ + 0.038 × C^2^ − 0.069 × D^2^ (3)

According to the results shown in Table A1, astragaloside I and astragaloside II were not detected in most test combinations, suggesting that the transformation of astragaloside I and astragaloside II into astragaloside IV was complete in the corresponding tests. As revealed by an analysis of the experimental results, the yield of astragaloside IV obtained after alkaline washing was slightly higher than the sum of astragaloside I, astragaloside II, and astragaloside IV. These results also demonstrated that saponins other than astragaloside I and astragaloside II in Radix Astragali could also be converted into astragaloside IV, as suggested in other studies [12,13].

#### 3.3.2. The ANOVA of Response Surface Methodology

**Figure 4:**
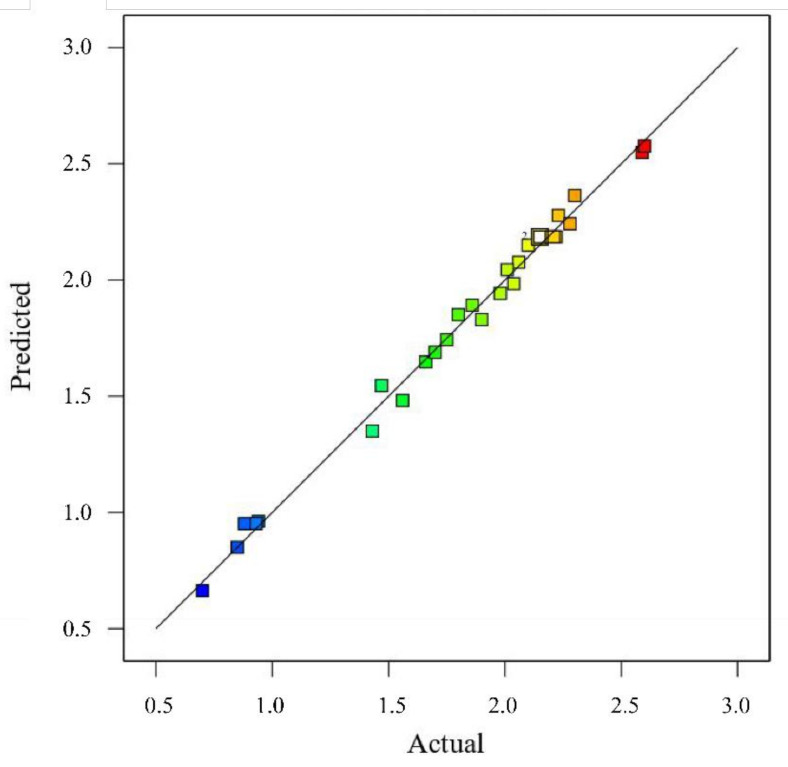
The comparison between the predicted and measured values of the yield of the astragaloside IV from Radix Astragali.

ANOVA in the software was conducted to evaluate the quadratic polynomial models [26]. A larger F-value and smaller *p*-value imply a more significant effect on the respective response methodology [27]. Table 4 lists the ANOVA results obtained for some important terms of the models. The adequacy of the model could be verified against the coefficient of determination (R^2^), the lack of fit, R^2^_adj_, AP, and CV tests. Among these parameters, R^2^ denotes the percentage of the variables as can be explained using the model. A higher R^2^ represents most of the variables, which can be explained by the model. The experimental data were consistent with the second-order polynomial equation. As for the parameters shown in Table 4, the R^2^ value was 0.9927. That is to say, the total variation reached only 0.73%, which could not be explained by the model. In this model, the value of R^2^ was sufficiently high to meet our requirement. In the meantime, R^2^_adj_ is as authoritative as R^2^ for the parameter R^2^_adj_ which is a modification of R^2^, with adjustment made to the number of descriptive terms. Different from R^2^, R^2^_adj_ only increases when the new term improves the model more significantly than expected [28]. It is better if the values of R^2^ and R^2^_adj_ are greater and closer to each other. From Table 4, it can be seen that the value of R^2^_adj_ of the model was 0.9853. The high value of R^2^_adj_ indicated that the model was significant. As indicated by the significance shown by the lack of fit test, the points were not adequately distributed around the model. Thus, the model could not be applied to predict the values of the independent variables. Therefore, the insignificance shown by the lack of fit test implied that the model could fit the data well [11]. In our model, all the “*p*-value prob > F” values of the lack of fit exceeded 10%, and they were insignificant. As indicated by these parameters, the model could fit the results well. AP was used to measure the signal-to-noise ratio, and a ratio greater than 4 was treated as desirable. In this model, the value of AP was 41.691, indicating that the signal was adequate. Moreover, the CV was supposed not to exceed 10% [29]. The value of the CV in our model was 3.52. In addition to the above-mentioned parameters, the predicted versus measured figure could also be used to demonstrate the suitability of the model. As shown in Figure 4, the predicted and actual measured experiment values were fitted almost in a straight line. As evidenced by this figure, the second-order polynomial regression model fit the experimental results well. Therefore, the models applied in this study could optimize the conditions for extracting astragaloside IV from Radix Astragali.

#### 3.3.3. The Impact of Different Factors on the Yield of Astragaloside IV

In Table 4, the linear and quadratic effects of the ammonia concentration, soaking time, and extraction time are shown to be significant (*p* < 0.05). The ammonia concentration with soaking time and solid–liquid ratio with soaking time were also found to be significant (*p* < 0.05).

**Figure 5:**
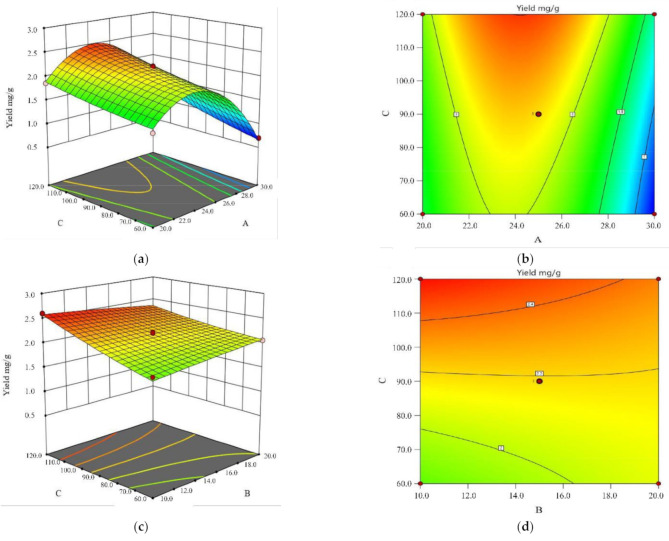
The 3D surfaces (**a,c**) and contour graphs (**b**,**d**) of the effects of ammonia concentration (A), solid–liquid ratio (B), and soaking time (C) on the yield of astragaloside Ⅳ.

Figure 5a shows the interaction between the ammonia concentration with soaking time. The yield of astragaloside IV first increased with the concentration of the ammonia and then decreased, which could be attributed to the treatment of alkaline wash that played a role in promoting the transformation of the astragalosides replaced with an acetyl group to astragaloside IV. Additionally, the higher alkalinity, the higher the degree of its transformation within the limits (ammonia concentration ≤ 25%). In this process, the production of astragaloside IV was significantly higher than the consumption of it. When alkalinity was sufficient, however, the saponins could also be effectively destroyed [15]. Thus, the yield of astragaloside IV treated with 30% ammonia was lower as compared to that treated with 25% ammonia. A longer soaking time provided more opportunities for other kinds of saponins to be transformed into astragaloside IV. As shown in Figure 5a,b, the yield of astragaloside IV reached its maximum when the ammonia concentration and soaking time approached 24% and 120 min, respectively.

Figure 5c shows the interaction between the solid–liquid ratio and soaking time. As the solid–liquid ratio was on the increase, the yield of saponins decreased. A higher solid–liquid ratio provided more opportunities for astragalus saponins and ammonia molecules, which could cause the destruction of saponins. As a phenomenon in flavonoids, glycosides could also produce aminoglycosides or aglycones as a result of alkali hydrolysis. The structure of those saponins could be undermined by alkali to varying degrees. As the soaking time was extended, the transformation from astragalosides replaced with an acetyl group to astragaloside IV was enhanced. The yield of the astragaloside IV significantly increased [15]. As shown in Figure 5c,d, the yield of astragaloside IV reached its maximum when the solid–liquid ratio and soaking time were approximately 10 and 120 min, respectively.

#### 3.3.4. Optimization and Validation Procedures

According to the constructed mode, the conditions were determined as follows. The ammonia concentration was 24.15%, the solid–liquid ratio was 1:10, the soaking time was 120 min, and the extraction time was 52 min. In this circumstance, the theoretical content of astragaloside IV was predicted to be 2.635 mg/g. After careful consideration, the ammonia concentration was set to 24%, the solid–liquid ratio was set to 1:10, the soaking time was set to 120 min, and the extraction time was set to 52 min. The experiment was repeated three times to verify the formulation. The average content of astragaloside IV was 2.621 ± 0.019 mg/g, which was basically consistent with the values as predicted using the constructed model and comparable with the extraction efficiency achieved by Yan, Mingming et al. [30].

### 3.4. The Changes in the Astragaloside IV Retention Rate Under Different Sterilization Conditions 

**Figure 6:**
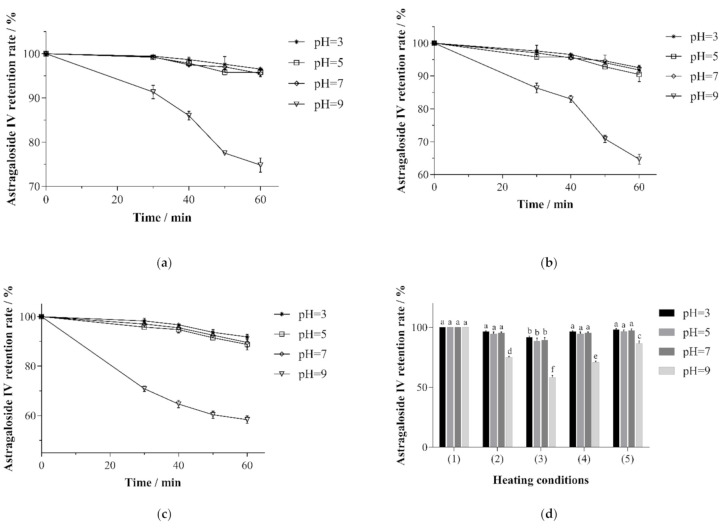
Changes in the retention rate of astragaloside IV of solutions under different sterilization conditions with different pH values. (**a**) The retention rate of astragaloside IV with different heating times at 85 °C. (**b**) The change of the retention rate of astragaloside IV with different heating times at 90 °C. (**c**) The change of the retention rate of astragaloside IV with different heating times at 95 °C. (**d**) The change of the retention rate of astragaloside IV with different heating conditions (group 1) at 100 °C for 0 min, heating at 100 °C for 15 min (group 2), heating at 100 °C for 30 min (group 3), heating at 105 °C for 15 min (group 4), and heating at 120 °C for 10 min (group 5). Different lowercase letter means differ significantly (*p* < 0.05).

Since the structure of astragaloside IV may be affected in an alkaline environment [15], the process of thermal sterilization could further accelerate this process, thus resulting in a significant decrease in the yield of astragaloside IV. As suggested by the results shown in Figure 6, the retention rate of astragaloside IV in the solutions at varying pH values was determined by different sterilization conditions. The solutions with pH values of 3.0, 5.0, 7.0, and 9.0 represented acidic (pH < 4.5), low-acidic (4.5 < pH < 7), neutral (pH = 7), and alkaline (pH *>* 7) food systems, respectively. As shown in Figure 6a, the thermal sterilization temperature was 85 °C. Astragaloside retention was weakened as the time of heat sterilization was extended. More specifically, the solutions with pH values of 3.0, 5.0, and 7.0 remained unchanged after 40 min of heating and showed a slight decrease (97.19 ± 1.91%~98.45 ± 1.40%) after 50–60 min of heating. The solution with a pH value of 9.0 dropped to 91.33 ± 1.53% when heated for 30 min. As heat sterilization was prolonged, the retention rate of astragaloside IV first declined to speed up and then slowed down to 74.83 ± 1.61% in 60 min, as shown in Figure 6b. The variation in Figure 6c is similar to that in Figure 6a, with the overall retention rate of astragaloside IV showing a decreasing trend. As shown in Figure 6b, the temperature was 90 °C. After 60 min of heating, the retention of astragaloside IV in solutions with varying pH values was 91.83 ± 1.01% (pH 3.0), 90.47 ± 2.17% (pH 5.0), 92.53 ± 0.74% (pH 7.0), and 64.67 ± 1.53% (pH 9.0). The temperature was 95 °C in Figure 6c. When the sterilization time was 60 min, the retention of astragaloside IV in solutions with varying pH values was 91.01 ± 2.32% (pH 3.0), 88.80 ± 2.21% (pH 5.0), 89.57 ± 1.91% (pH 7.0), and 58.37 ± 1.10% (pH 9.0). For Figure 6d, the process at a high temperature and with a short sterilization time was performed. After heating 100°C/15 min, 105°C/15 min, and 120°C/15 min, there was no significant difference (*p >* 0.05) between the retention rate of astragaloside IV in different pH. In contrast, the retention rate of astragaloside IV at pH 9.0 was reduced significantly. After being heated at 120 °C for 10 min, however, the retention rate of astragaloside IV was 86.67 ± 2.08%, which was sufficient to preserve astragaloside IV in the alkaline solution better than in other germicidal conditions. In the meantime, astragaloside IV is the final form of transformation for astragalus saponins [13]. After heat sterilization, the astragaloside IV extracted by the alkaline solution could not be converted back to other saponins. In the solution, there were no other astragalus saponins detected (astragaloside I and astragaloside II).

In this paper, the stability of astragaloside in the process of thermal sterilization was first studied. Regarding Chinese herbal medicines (CHMs), it is common for the transformation of chemical constituents to occur as a result of high temperature, high humidity, and either high or low pH values. In this paper, astragaloside IV was extracted using an alkaline solution, and the acidity of the subsequent processing environment could vary according to the actual situation. Due to the potential of transformation between astragalus saponins, the stability of astragaloside is worth paying particular attention to. Up to now, however, there have been few studies focusing on the stability of astragalus saponins in different acidity levels at room temperature, and saponins lack stability in the process of thermal sterilization. For example, Mei X.D. et al. [15] made an attempt to extract different bases, but they ignored the subsequent stability. Additionally, no consideration was given to the potential damage caused by temperature to astragaloside IV. According to these experimental results, a higher sterilization temperature and a longer sterilization time were more damaging to astragaloside IV. Moreover, astragaloside IV can maintain stability and is also more resistant to heat sterilization in acidic, low-acidic, or neutral solution environments than in an alkaline solution, which is conducive to food processing.

### 3.5. Changes in the Astragaloside IV Retention Rate under Different Storage Conditions 

**Figure 7:**
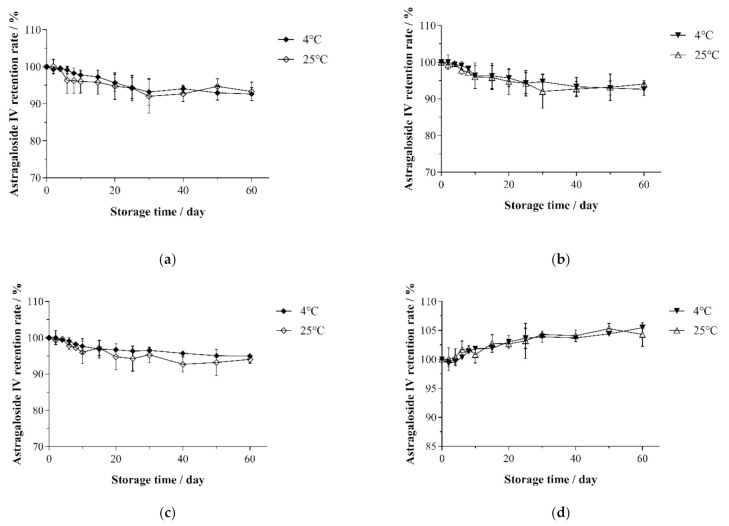
Changes in the retention rate of astragaloside IV in solutions with different pH values: (**a**) pH 3.0, (**b**) pH 5.0, (**c**) pH 7.0, and (**d**) pH 9.0 during the storage at 4 or 25 °C.

For example, after 40 min of heating at 85 °C, the retention rate of astragaloside IV in the solution with different pH values (pH 3.0, 5.0, 7.0, and 9.0) varied within 60 days of storage at room temperature. As shown in Figure 7a–c, the retention rate of astragaloside IV in acidic (pH 3.0), low-acidic (pH 5.0), and neutral (pH 7.0) solutions decreased in the first 30 days of storage to 92.58 ± 3.75%, 93.33 ± 3.50%, and 95.92 ± 1.52%, respectively. In contrast, the value dropped slightly to 92.98 ± 1.97%, 93.33 ± 1.50%, and 94.50 ± 0.91%, respectively during 30–60 days. From Figure 7d, it can be seen that the yield of astragaloside IV in the alkaline (pH 9.0) solution increased at a slow pace during storage and reached 104.88 ± 1.46% after 60 days of storage. Since astragaloside IV is the basic skeleton structure of the astragaloside, it is a common transformation product for other astragalosides such as astragaloside I, astragaloside II, isoastragaloside II, astragaloside I, isoastragaloside I, acetylastragaloside I, and malonylastragaloside I [15]. Additionally, Figure 7 reveals no significant difference in the effect caused by cold storage and room temperature storage on astragaloside. In this study, the optimal extraction process was conducted to obtain an astragaloside IV extract. After thermal sterilization, the stability of astragaloside IV was assessed. Different from the work Chu C. et al. [13], an astragaloside standard was applied, and several Radix Astragali medicinal materials were taken as raw materials. After extraction was performed at room temperature, adjustment was made to the acidity of the solution to determine the change in astragaloside IV content under acidic, neutral, and alkaline environments. This was mainly attributed to the failure in completing the transformation of astragalus saponins in the early stage. Furthermore, Zheng N. et al. [16] conducted a study to demonstrate that astragaloside I is more susceptible to temperature and acidity than astragaloside I, thus confirming the above conjecture. Regarding food processing, the appropriate storage conditions can be determined according to the need to ensure product safety.

## 4. Conclusions

In recent years, Radix Astragali has been increasingly used in food processing, which makes it necessary to improve the yield of astragaloside IV during extraction. In this study, it was found out that both astragaloside I and astragaloside II were transformed completely to astragaloside IV under the optimal conditions. These conditions could be used to replace the traditional organic solvent reflux extraction method and are applicable in food processing because of their efficiency, low energy consumption, and non-toxicity. It was also found out that astragaloside IV was more stable in acidic, low-acidic, and neutral foods than in alkaline foods during sterilization. In alkaline foods, a high temperature and a short sterilization time can enhance the stability of astragaloside IV. Moreover, for acidic, low-acidic, and neutral foods, more thermal sterilization conditions can be selected. Moreover, it was discovered that the astragaloside IV obtained under the extraction conditions applied in this study could maintain a high stability during storage and facilitate food processing. In food processing, sterilization conditions can be determined on the basis of the experimental results obtained this study. For our future study, the focus will be placed on the changes in astragaloside IV during food processing for adaptation to more food processing scenarios, and physiological function will be analyzed.

## Figures and Tables

**Table 1:** The factors and levels for the response surface methodology design.

The Factors	Level		
	−1	0	1
Ammonia concentration (A) (%, *v/v*)	20	25	30
Solid–liquid ratio (B) (mL/g)	10:1	15:1	20:1
Soaking time (C) (min)	60	90	120
Extraction time (D) (min)	0	30	60

**Table 2:** MRM parameters of astragaloside IV.

	Qualitative Ion Pair	Quantitative Ion Pair	Cone Hole Voltage/V	Collision Energy/eV
Astragaloside I	891.5 > 891.5	891.5 > 891.5	13	10
Astragaloside II	849.4 > 849.4	849.4 > 849.4	14	9
	827.5 > 827.5		14	10
	827.5 > 143		14	11
Astragaloside IV	785.4 > 473.39	785.4 > 143.11	14	12
	785.4 > 143.11		14	10

**Table 3:** Quantitative validation.

Parameters	Substances		
	Astragaloside I	Astragaloside II	Astragaloside IV
Calibration curve	y = 400.12x + 1309.5	y = 655.11x + 1353.9	y = 1047.7x + 197.46
Test range (μg/mL)	0.083–2.67	0.042–1.33	0.026–0.83
r^2^	0.9991	0.9935	0.9997
LOD (μg)	0.006	0.003	0.002
LOQ (μg)	0.024	0.015	0.009
Intra-day RSD (%) (*n* = 5)	1.5	3.7	1.4
Inter-day RSD (%) (*n* = 5)	1.8	1.2	1.1
Recoveries (%)	94.1 ± 4.3	98.4 ± 5.8	96.5 ± 1.9

y: peak area ratio of the analyte/internal standard; x: concentration of analyte (μg/mL).

**Table 4:** Results of the ANOVA to the response surface quadratic model for the yield of astragaloside IV.

Content of Astragaloside IV	Sum of Squares	Degrees of Freedom	Mean Square	F-Value	*p*-value
Model	7.71	14	0.55	135.37	<0.0001
A	1.52	1	1.52	373.33	<0.0001
B	8.00 × 10^−4^	1	8.00 × 10^−4^	0.20	0.6642
C	0.80	1	0.80	196.77	<0.0001
D	0.099	1	0.099	24.42	0.0002
AB	6.25 × 10^−4^	1	6.25 × 10^−4^	0.15	0.7010
AC	0.029	1	0.029	7.10	0.0185
AD	6.40 × 10^−3^	1	6.40 × 10^−3^	1.57	0.2304
BC	0.053	1	0.053	13.00	0.0029
BD	5.77 × 10^−3^	1	5.77 × 10^−3^	1.42	0.2533
CD	8.1 × 10^−3^	1	8.1 × 10^−3^	1.99	0.1801
A2	4.81	1	4.81	1181.04	<0.0001
B2	9.87 × 10^−4^	1	9.87 × 10^−4^	0.24	0.6301
C2	9.33 × 10^−3^	1	9.33 × 10^−3^	2.29	0.1523
D2	0.031	1	0.031	7.50	0.0160
Residual	0.057	14	4.07 × 10^−3^		
Lack of Fit	0.052	10	5.25 × 10^−3^	4.64	0.0761
Pure Error	4.52 × 10^−3^	4	1.13 × 10^−3^		
Cor Total	7.77	28			
R-Squared			0.9927		
Adj R-Squared			0.9853		
C.V. %			3.52		
Adequate Precision			41.691		

## Sample Availability

Samples of the astragaloside I, astragaloside II, and astragaloside IV standard compounds are available from the authors.

## Appendix A

**Table A1:** The experiment results of response surface methodology. Data were expressed as mean ± standard deviation (SD) (*n* = 3).

Treatment Number	Ammonia Concentration (A) (%, *v/v*)	Solid–Liquid Ratio (B) (mL/g)	Soaking Time (C) (min)	Extraction Time (D) (min)	Content of Astragaloside IV (mg/g)	Astragaloside Ⅰ (mg/g)	Astragaloside Ⅱ (mg/g)
1	25	10	90	0	2.012 ± 0.073	Not detected	Not detected
2	25	20	120	30	2.301 ± 0.011	Not detected	Not detected
3	25	10	60	30	1.904 ± 0.023	Not detected	Not detected
4	25	15	120	0	2.235 ± 0.084	Not detected	Not detected
5	30	15	60	30	0.701 ± 0.036	0.107 ± 0.005	0.142 ± 0.010
6	25	20	90	0	2.049 ± 0.042	Not detected	Not detected
7	25	20	90	60	2.283 ± 0.086	Not detected	Not detected
8	25	15	90	30	2.215 ± 0.061	Not detected	Not detected
9	30	15	120	30	1.436 ± 0.021	Not detected	0.086 ± 0.002
10	30	20	90	30	0.938 ± 0.025	0.067 ± 0.004	0.103 ± 0.003
11	20	15	60	30	1.473 ± 0.077	Not detected	0.079 ± 0.002
12	25	15	90	30	2.157 ± 0.113	Not detected	Not detected
13	30	15	90	0	0.851 ± 0.032	0.076 ± 0.003	0.129 ± 0.004
14	30	10	90	30	0.940 ± 0.023	0.071 ± 0.002	0.116 ± 0.003
15	25	15	60	60	1.981 ± 0.049	Not detected	Not detected
16	20	20	90	30	1.702 ± 0.026	Not detected	Not detected
17	25	20	60	30	2.063 ± 0.036	Not detected	Not detected
18	25	10	120	30	2.602 ± 0.072	Not detected	Not detected
19	25	15	90	30	2.151 ± 0.034	Not detected	Not detected
20	25	15	90	30	2.220 ± 0.065	Not detected	Not detected
21	20	15	90	0	1.566 ± 0.073	Not detected	0.067 ± 0.002
22	20	15	90	60	1.757 ± 0.029	Not detected	Not detected
23	20	15	120	30	1.861 ± 0.016	Not detected	Not detected
24	25	15	90	30	2.205 ± 0.035	Not detected	Not detected
25	25	10	90	60	2.102 ± 0.039	Not detected	Not detected
26	30	15	90	60	0.882 ± 0.026	0.069 ± 0.003	0.125 ± 0.002
27	25	15	120	60	2.590 ± 0.108	Not detected	Not detected
28	20	10	90	30	1.660 ± 0.025	Not detected	0.062 ± 0.002
29	25	15	60	0	1.801 ± 0.049	Not detected	Not detected
